# A Behavioral and fNIRS Comparative Study of Gender and Task Differences in Mental Rotation Among Primary Students

**DOI:** 10.1002/brb3.70358

**Published:** 2025-03-23

**Authors:** Dandan Wu, Jinfeng Yang, Zhi Hong Wan, Yining Shen, Qianming Liu, Jinghui Zhang, Simin Cao, Hui Li

**Affiliations:** ^1^ Department of Early Childhood Education The Education University of Hong Kong Hong Kong China; ^2^ Department of Medical Imaging Henan Provincial People's Hospital and People's Hospital of Zhengzhou University Zhengzhou China; ^3^ Key Laboratory of Modern Teaching Technology Ministry of Education Xi'an People's Republic of China; ^4^ Shanghai Institute of Early Childhood Education Shanghai Normal University Shanghai China; ^5^ School of Education Macquarie University Sydney New South Wales Australia

**Keywords:** behavioral measures, fNIRS, gender effect, mental rotation, task effect, Type II error

## Abstract

**Purpose:**

This study investigated the sex and task effects in mental rotation (MR) among Chinese primary school students, employing behavioral assessments and functional near‐infrared spectroscopy (fNIRS) for a comprehensive analysis.

**Method:**

The sample consisted of 62 Grade 4 and 5 students aged between 9.58 and 11.33 (*M*_age_ = 10.604, SD = 0.35). Participants completed two MR tasks: MR1, which required the rotation of 24 figures, and MR2, which involved the rotation of 16 English letters. fNIRS was employed to measure neural activation in specific brain areas, and behavioral performance was assessed alongside brain activity.

**Findings:**

Behavioral data revealed no significant effects of sex or task type on the performance of MR1 or MR2. However, the fNIRS results demonstrated notable sex and task effects. Specifically, boys exhibited significantly higher brain activation in Brodmann Areas (BAs) 6, 9, and 46 than girls. In contrast, girls showed significantly more activation in BA 46 during the MR1 figure rotation task. This suggests that while behavioral tasks showed no differences, neuroimaging revealed underlying neural disparities.

**Conclusion:**

The discrepancy between behavioral and fNIRS findings reveals a tendency in behavioral studies to accept a false negative, resulting in Type II errors. While neuroimaging data indicate substantial differences not reflected in behavioral measures, this challenges the reliability of null results typically associated with traditional behavioral paradigms. This study highlights the critical need to integrate neuroimaging techniques to understand cognitive processes better. Furthermore, it emphasizes the importance of reevaluating conventional interpretations of behavioral data to ensure a more comprehensive view of mental function.

## Graphical abstract

Regions that show the Task and Group × Task interaction main effect in the mental rotation task marked on MNI space: Region G (BA 46) contains Channels 30 and 34. The difference found in the Task and Group × Task main effect is circled in red.

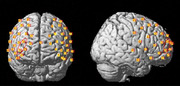

## 1 Introduction

Mental rotation (MR) is the cognitive ability to manipulate and visualize objects mentally from various perspectives, enabling individuals to imagine how objects would appear when rotated in space. This skill is essential for numerous spatial reasoning tasks, including solving puzzles, navigating environments, and interpreting diagrams or maps. First introduced by Shepard and Metzler (1971), MR has since been recognized as an innate dynamic ability (Uttal et al. 2013). The interplay of sex and task effects on MR abilities, particularly during the critical developmental period from ages 0 to 12, remains a contentious topic (Tolar et al. 2009). Certain studies have highlighted sex differences in MR, attributing these variations to biological factors like hormonal influences (Baenninger and Newcombe 1989; Courvoisier et al. 2013; Steele and Aronson 1995; Voyer et al. 1995). Conversely, other research emphasizes the impact of specific tasks on MR performance, linking outcomes to time constraints (Voyer 2011) and task material stereotypes (Rahe et al. 2020). Recent studies have increasingly focused on the dynamic relationship between sex and task effects on MR across different age groups in children, yielding mixed results (Fargier et al. 2022; van Tetering et al. 2019; Voyer et al. 2020). With advancements in neuroimaging techniques, we can reassess MR's conflicting behavioral evidence and gain insights into the underlying neuropsychological mechanisms. Therefore, our study aims to explore the combined effects of sex and task factors on MR performance among Chinese primary students, employing both behavioral assessments and functional near‐infrared spectroscopy (fNIRS). By aligning our behavioral observations with neuroimaging findings, we hope to contribute meaningfully to the ongoing discussion surrounding sex and task effects in MR.

### 1.1 Sex Differences in MR

The issue of sex differences in MR capabilities has been a fascinating subject of study, with sex identified as one of the critical contributors to individuals' performance (Hyde 2005). Several studies have shown that males typically outstrip their female counterparts regarding such tasks (Voyer et al. 1995). This differential performance rate in MR exercises between the two sexes was hailed as a significant cognitive diversity supported by multiple meta‐analytic findings (Hyde 2005). The significance of this sex disparity in MR proficiency propelled numerous scientific inquiries aimed at deciphering the inherent causes. Scholars frequently proposed myriad determinants, including individual spatial abilities, regarded as a form of skill (Lohman 1986). Nevertheless, other studies underlined the adaptability of MR abilities, which evidently could be improved through training (Uttal et al. 2013). This adaptability implies that the observed sex differences might not solely be due to individual spatial ability, thus necessitating further exploration of supplementary factors.

Investigations seeking to explain these differences have focused on various components ranging from biological and sociocultural to measurement variables. The biological factors typically considered include brain structure (Hugdahl et al. 2006; Jordan et al. 2002) and hormone levels (Berenbaum et al. 1995; Courvoisier et al. 2013). Sociocultural factors feature prominently, too, and encapsulate variables like childhood activities (Cherney and Voyer 2010) and sex‐role identification (Wraga et al. 2006). Additionally, measurement variables such as item types (Voyer and Hou 2006) and item familiarity (Bethell‐Fox and Shepard 1988; Neuburger et al. 2011) were also investigated as potential influencers of sex disparities in MR.

Initially, van Tetering et al. (2019) study on 3D MR abilities in school children (7–12 years) found a sex disparity, with boys scoring higher on the MR Task–Children (MRT–C) and exhibiting a positive correlation between rotational performance and mathematics achievement. Voyer et al. (2020) implemented eye‐tracking in a large‐scale study comparing male and female performance on MR tasks the following year. Men consistently outperformed women, though all participants performed better when human figures were utilized over blocks. Later, Fargier et al. (2022) pursued response‐time discrepancies between sexes in a similar task with undergraduate students, finding men responding more rapidly and physical activity potentially affecting results. Rahe and Jansen (2022)’s findings also showed that males generally outperformed females when using sex‐stereotyped objects, emphasizing the need to consider the nature of the test due to sex performance divergences under differing conditions. However, these behavioral studies overlooked the potential value of neuroimaging evidence in assessing sex‐based performance variations in MR tasks.

### 1.2 Task Differences in MR

The existing studies have demonstrated that men notably outperformed women on the paper‐and‐pencil MR test (MRT) (Vandenberg and Kuse 1978). However, findings became ambiguous when chronometric tests were used, usually involving two objects placed adjacently with one rotated about the other (Jansen‐Osmann and Heil 2007). In these tests, marked sex differences favoring men were only observed in polygons, not cube figures, letters, animal drawings, or abstract figures. Interestingly, women enhanced performance when using human figures instead of cube figures (Alexander and Evardone 2008; Doyle and Voyer 2018; Jansen and Lehmann 2013).

The type of objects to be rotated might also significantly impact sex performance. For instance, two distinct MRTs were developed using abstract cube and pellet figures (Ruthsatz et al. 2014) and stereotypically male and female objects (Ruthsatz et al. 2015). An adult study found that using the test with cube and pellet figures resulted in sex differences that advantaged men, regardless of stimulus material (Rahe et al. 2020). However, when using more stereotypical items (e.g., hammer, locomotive, and revolver for males, and hairbrush, doll, and hand mirror for females), the findings showed an interaction between the type of stimulus and sex. Favorable sex differences for men were only observed for male‐stereotyped items but not female‐stereotyped ones.

In a recent study, a stereotyped psychometric MRT was employed, and it was proposed that more research was needed to ascertain if the observed sex differences favoring men with male‐stereotyped items persist in a chronometric version of this test (Ruthsatz et al. 2019). Intriguingly, in this study involving 10‐year‐old children, conducted with mobile computers utilizing touchscreens, no noteworthy interactions between sex and material for accuracy were detected. However, this controversial behavioral finding has triggered our interest in examining the effects of sex‐task interaction with fNIRS in this study. In particular, we utilized a chronometric task based on a “same/different” choice format to analyze cognitive processes involved in MR. This approach was chosen to establish controlled conditions that facilitate a clearer understanding of how participants discern between varying rotational differences, particularly in scenarios where more significant discrepancies are presented among the options.

### 1.3 Integrating Behavioral and Neuroimaging Methods in MR Studies

Emerging evidence indicates the existence of sex differences in brain activation during MR tasks, often with males having an advantage. For example, certain studies, such as Jordan et al. (2002), reported dissimilar cortical activation patterns in males and females, even with comparable task performance levels. This suggests that different problem‐solving strategies may be employed based on sex. Hugdahl et al. (2006) furthered this viewpoint, concluding that females resort to categorical processing while males utilize coordinate processing in visuospatial tasks. Moreover, Neubauer et al. (2010) found sex differences exclusively in two‐dimensional (2D) representations of three‐dimensional (3D) objects but not in actual 3D or virtual reality presentations. Their research indicated that males exhibited increased neural efficiency post‐training. Semrud‐Clikeman et al. (2012) also reported that men displayed more robust activation of visual attention networks than women during MR tasks. This evidence insinuates that men may lean towards more automatic processes while women implement a top‐down approach.

Interestingly, Jaušovec and Jaušovec (2012) discovered sex disparities in brain activity only in high‐performance participants, indicating that training could alter these differences. In their study, females within the trained group exhibited decreased frontal brain activity and augmented parietal activation post‐training, but these changes were not seen in the control group. These established differences in visuospatial abilities based on sex were also seen to be influenced by nationality and cognitive processing speed, as highlighted in Jansen et al. (2016), where it was found that German students outperformed Omani students in MR tasks. Therefore, training, nationalities, and cultures might also contribute to the sex and task differences in MR performance.

Frick et al. (2013) assessed the MR abilities of 3‐ to 5‐year‐olds using an innovative puzzle paradigm, allowing for an evaluation of younger children with tasks typically reserved for older age groups. Their findings revealed a marked improvement in children's ability to select the correct asymmetrical ghost figure as they aged, with only 10% of 3‐year‐olds correctly identifying it in a 3D task, compared to an impressive 95% of 5‐year‐olds, a trend mirrored in 2D tasks. Newcombe (2020) reviewed the development of sex‐related differences in spatial skills, emphasizing that these differences increase with age and are influenced by task parameters and cultural contexts. Although training can enhance spatial skills, current interventions have not eliminated the male advantage, highlighting a crucial need for continued research into the cognitive and neural foundations of spatial abilities across diverse populations. Further supporting this, cross‐sectional studies indicate that the ability to form mental maps enhances throughout childhood, reaching adult proficiency by early adolescence, with notable individual variations. A recent longitudinal analysis by Brucato et al. (2022) demonstrated that performance on spatial tasks, especially in route integration and MR, becomes more stable by age 12, suggesting that older children display increased consistency in these critical cognitive skills over time.

In addition, stereotype threat theory posits that awareness of negative stereotypes about one's group can impair cognitive performance, especially in stereotype‐relevant domains (Steele and Aronson 1995). In MR, a task often associated with masculine stereotypes regarding spatial abilities, women may experience performance decrements due to the anxiety and self‐doubt evoked by this awareness (Cadinu et al. 2005). This decrement reflects not a deficit in ability but a situational response to the pressure of potentially confirming the negative stereotype. Cultural context also plays a role, with stereotype threat effects potentially amplified in cultures with rigid gender roles and stronger associations between spatial skills and masculinity. Research has demonstrated the efficacy of interventions to mitigate stereotype threat, such as promoting positive self‐affirmation and presenting counter‐stereotypical role models (Kiefer and Sekaquaptewa 2007). Further supporting the role of implicit stereotypes, Guizzo et al. (2019) found that implicit gender‐spatial stereotyping (IGSS), measured via an implicit association test (IAT), moderated the effect of stereotype‐related instructions on men's MR performance. In their study (*N* = 143), men with stronger IGSS, associating men with spatial abilities, performed worse after receiving stereotype‐nullifying instructions than stereotypical or lower IGSS. This suggests that the incongruence between internalized stereotypes and external messages can negatively impact performance. While men generally outperformed women, replicating prior findings, no such interaction was found for women. These findings underscore the importance of considering implicit stereotypes as moderators of stereotype threat effects and highlight the potential for unintended consequences when challenging ingrained stereotypes. Therefore, future research on stereotype threat in spatial cognition should incorporate implicit measures, as Guizzo et al. (2019) advocate.

These findings underlined the importance of examining sex and task differences in MR performance with different populations, approaches, and contexts. However, there is still a scarcity of research on adopting neuroimaging methods to investigate the task differences in MR performance. Little is known about the extent to which the findings generated by behavioral and neuroimaging methods on MR performance are consistent or inconsistent. The study will fill these gaps by comparing the behavioral and fNIRS findings on sex and task differences in MR of Chinese primary students. More specifically, the following questions will be investigated:

1. Are there any significant sex differences in primary students’ MR performance assessed through behavioral and fNIRS methods?
2. Are there any significant task differences in primary students’ MR performance assessed through behavioral and fNIRS methods?
3. Are there significant effects of sex‐task interaction on primary students’ MR performance assessed through behavioral and neuroimaging methods?

In particular, we examined the following hypotheses in this study:

*H1*: There are significant sex differences in primary students’ MR performance as assessed through behavioral and fNIRS methods.
*H2*: There are significant task differences in primary students’ MR performance as assessed through behavioral and fNIRS methods.
*H3*: There are significant interaction effects between sex and task in primary students’ MR performance as assessed through behavioral and neuroimaging methods.

## 2 Method

### 2.1 Participants

Whole class sampling of Grades 4 and 5 was conducted in a primary boarding school dedicated to left‐behind children in Henan Province, central China, resulting in 64 right‐handed Chinese students. These children belong to families with low socioeconomic status (SES), as their parents have migrated to major cities for work, leaving them in the care of the school for their primary education. The participants had been taught the English alphabet by Grade 3, a prerequisite for their involvement in this study. All parents or guardians provided written consent, and they were verbally informed about the study's purpose and the safety measures related to the fNIRS experiments, which received approval from the ethics committee of the first author's university. Two participants could not complete the experiments and were subsequently excluded from the study, resulting in a final sample of 62 students aged between 9.58 and 11.33 years (*M*_age_ = 10.604, SD = 0.35).

### 2.2 MR Tasks and Procedure

**FIGURE 1:**
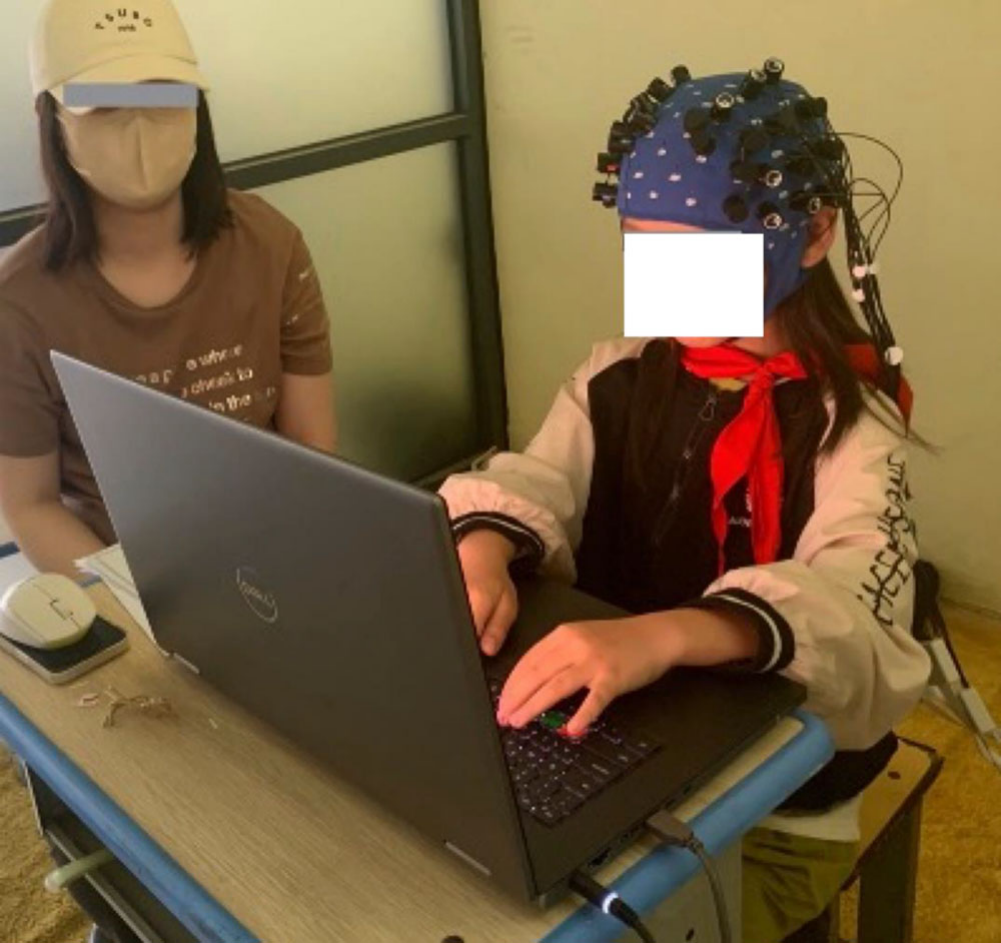
Experimental environment. The subject performed the mental rotation (MR) task during functional near‐infrared spectroscopy recording.

A modified version of the MR task proposed by Jansen et al. (2013) was utilized in this study, incorporating two stimulus tasks, including the “animal figures” (MR Task 1) and the “letters” condition (MR Task 2). MR1 contained black‐and‐white figures of 24 animals (dog, horse, monkey, pig, and tiger) used in Snodgrass and Vanderwart (1980). MR2 presented 15 English letters (e, F, g, j, k, L, l, n, P, q, R, r, s, S, and Z). Figure 1 displays the experimental environment. The test and target stimuli were presented on ePrime on a 16‐in. Dell notebook, and the first author sat beside the participants and guided them with the task. One stimulus remained unrotated, representing the target stimulus on the left side. In contrast, the other was rotated to angles such as 45°, 90°, or 135° (clockwise or counterclockwise), representing the test stimulus on the right side. We chose 2D stimuli (letters and figures) for this study because the samples were left‐behind children without experience with 3D stimuli.

**FIGURE 2:**
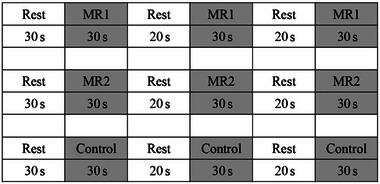
The MR task paradigm.

During the training and testing phases, each child participated in a session in which they were presented with a pair of stimuli. They were asked to determine as quickly as possible whether the stimuli were “different” (e.g., mirror‐reversed) or “identical.” Each child was instructed to press a yellow button to indicate “different” stimuli and a blue button to denote “identical” stimuli. Verbal feedback from the experimenter was provided only during the training trials, while no feedback or additional instructions were given during the formal testing trials. The dependent measures comprised the total number of correct responses across all phases. For more details on the formal testing session, please refer to Figure 2.

### 2.3 Data Acquisition

**FIGURE 3:**
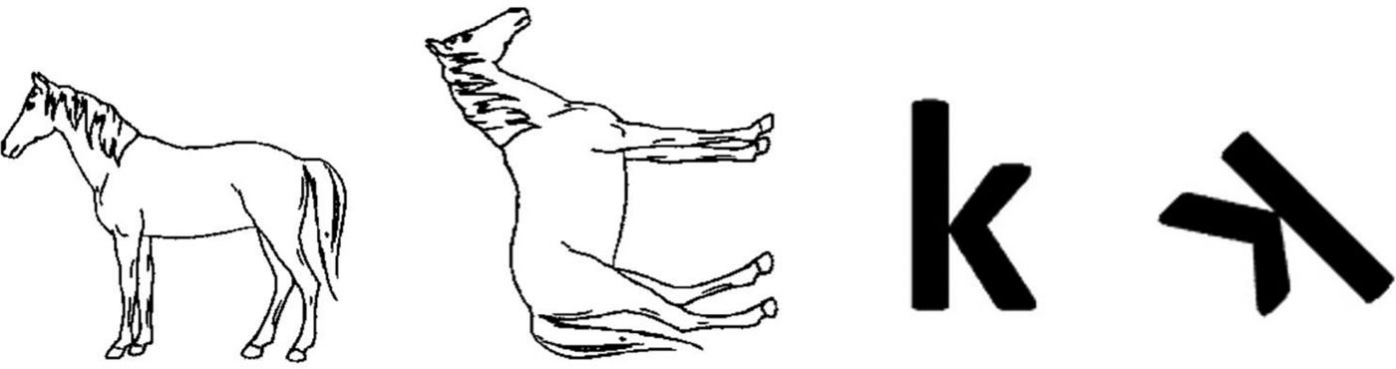
Stimulus in the formal test (animal and letter).

**FIGURE 4:**
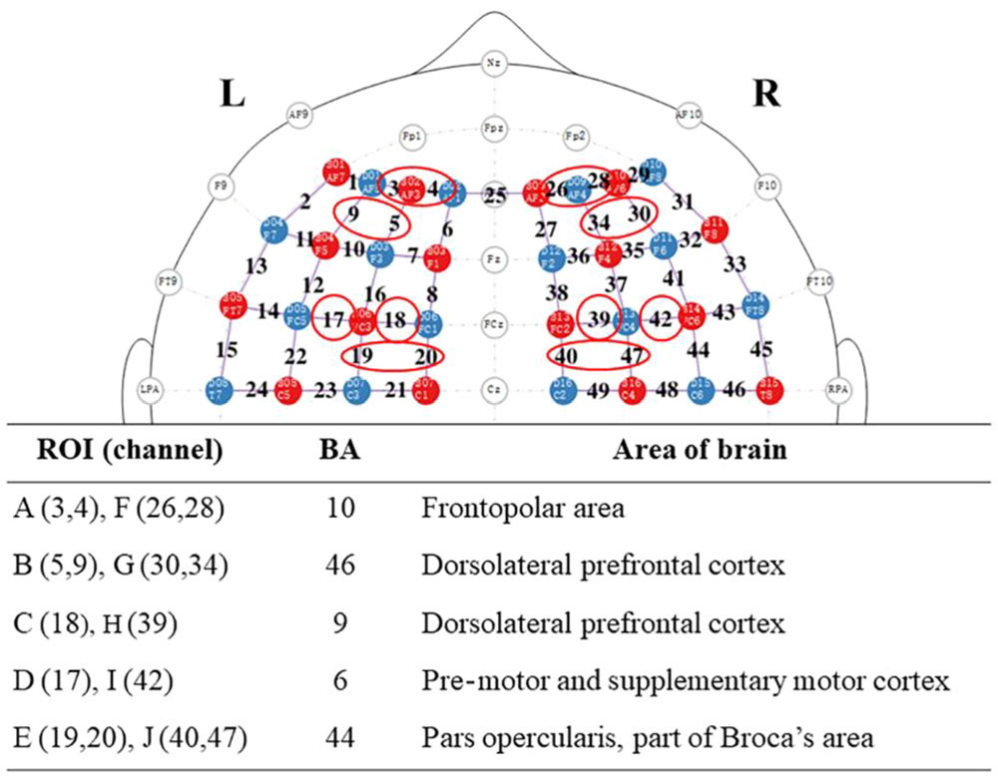
Localization of regions of interest.

In this study, we employed the NIRSport2 system (NIRx Medical Technologies, Germany), a portable and user‐friendly fNIRS instrument, to simultaneously measure changes in the concentration of oxygenated hemoglobin (HbO), deoxygenated hemoglobin (HbR), and total hemoglobin (HbT) in participants. We used two wavelengths in the near‐infrared range—760 and 850 nm—to assess changes in optical density (OD), which were then converted into HbO and HbR concentration changes using the modified Beer–Lambert law (Delpy et al. 1988). To ensure spatial accuracy, we utilized the EEG 10–20 electrode positioning system (Jurack et al. 2007) to properly position the light sources and detectors on the participants' heads. These emitters and detectors were secured using a custom‐built headcap (Figure 3). Each participant was equipped with two 4 × 4 probe holders, comprising 16 NIRS light emitters and 16 detectors spaced approximately 3 cm apart, resulting in a total of 48 data channels sampled at 5.1 Hz (see Figure 4). These probe sets were primarily placed in the left and right prefrontal regions.

We recorded the probe and channel positions on each participant's scalp to capture anatomical brain information using a 3D digitizer. We then registered the fNIRS probe positions to the MNI space via a probabilistic estimation process using NIRS_SPM. The Brodmann atlas was employed to determine the regions associated with each channel; if a channel were located in areas A, B, and C, the NIRS_SPM toolbox would generate a list (see Table 1) outlining the corresponding Brodmann area (BA) and the probability of the scalp position being found in those areas. For subsequent analysis, we selected channels located in BAs with a probability greater than 0.7.

**TABLE 1:** Channel location list.

Channel	Anatomical label from the Brodmann atlas	Probability
1, 29	10—Frontopolar area	0.38554
	11—Orbitofrontal area	0.064257
	46—Dorsolateral prefrontal cortex	0.35743
	47—Inferior prefrontal gyrus	0.19277
2, 31	45—pars triangularis Broca's area	0.0076336
	45—pars triangularis Broca's area	0.0076336
	Inferior prefrontal gyrus	0.47328
3, 28	10—Frontopolar area	0.84477
	11—Orbitofrontal area	0.15523
4, 26	10—Frontopolar area	0.99278
	46—Dorsolateral prefrontal cortex	0.0072202
5, 34	10—Frontopolar area	0.12397
	10—Frontopolar area	0.12397
6, 27	9—Dorsolateral prefrontal cortex	0.51639
	10—Frontopolar area	0.2541
	46—Dorsolateral prefrontal cortex	0.22951
7, 36	9—Dorsolateral prefrontal cortex	0.47964
	46—Dorsolateral prefrontal cortex	0.52036
8, 38	46—Dorsolateral prefrontal cortex	0.52036
	9—Dorsolateral prefrontal cortex	0.44492
9, 30	10—Frontopolar area	0.14729
	45—pars triangularis Broca's area	0.015504
	46—Dorsolateral prefrontal cortex	0.83721
10, 35	45—pars triangularis Broca's area	0.70566
	46—Dorsolateral prefrontal cortex	0.29434
11, 32	38—Temporopolar area	0.0033445
	45—pars triangularis Broca's area	0.54515
	46—Dorsolateral prefrontal cortex	0.36455
	47—Inferior prefrontal gyrus	0.086957
12, 41	44—pars opercularis, part of Broca's area	0.055738
	45—pars triangularis Broca's area	0.94426
13, 33	38—Temporopolar area	1
14, 43	6—Premotor and supplementary motor cortex	0.21405
	38—Temporopolar area	0.1505
	44—pars opercularis, part of Broca's area	0.036789
	48—Retrosubicular area	0.59866
15, 45	21—Middle Temporal gyrus	1
16, 37	9—Dorsolateral prefrontal cortex	0.12917
	44—pars opercularis, part of Broca's area	0.22917
	45—pars triangularis Broca's area	0.51667
	46—Dorsolateral prefrontal cortex	0.125
17, 42	6—Premotor and supplementary motor cortex	0.19929
	44—pars opercularis, part of Broca's area	0.75445
	45—pars triangularis Broca's area	0.046263
18, 39	6—Premotor and supplementary motor cortex	0.12185
	8—Includes Frontal eye fields	0.084034
	9—Dorsolateral prefrontal cortex	0.79412
19, 47	4—Primary motor cortex	0.055118
	6—Premotor and supplementary motor cortex	0.9252
	9—Dorsolateral prefrontal cortex	0.019685
20, 40	6—Premotor and supplementary motor cortex	0.77241
	8—Includes frontal eye fields	0.22759
21, 49	3—Primary somatosensory cortex	0.19048
	3—Primary somatosensory cortex	0.19048
	6—Premotor and supplementary motor cortex	0.20238
22, 44	6—Premotor and supplementary motor cortex	0.23871
	22—Superior temporal gyrus	0.032258
	43—Subcentral area	0.6871
	48—Retrosubicular area	0.041935
23, 48	1—Primary somatosensory cortex	0.34448
	2—Primary somatosensory cortex	0.23077
	3—Primary somatosensory cortex	0.16722
	4—Primary motor cortex	0.006689
	43—Subcentral area	0.25084
24, 46	21—Middle temporal gyrus	0.4717
	22—Superior temporal gyrus	0.5283

Based on previous studies (Wu et al. 2020; Yang et al. 2020) suggesting that BAs 6, 9, 10, 44, and 46 are involved in MR processing, we defined ten regions of interest. As illustrated in Figure 4, these regions are as follows: Region A includes Channels 3 and 4; Region B comprises Channels 5 and 9; Region C corresponds to Channel 18; Region D encompasses Channel 17; Region E includes Channels 19 and 20; Region F corresponds to Channels 26 and 28; Region G comprises Channels 30 and 34; Region H corresponds to Channel 39; Region I includes Channel 42; and Region J consists of Channels 40 and 47. Precisely, Regions A and F, located in the frontopolar area, primarily correspond to BA 10; Regions B and G, located in the dorsolateral prefrontal cortex (DLPFC), correspond to BA 46; Regions C and H also reside in the DLPFC, corresponding to BA 9; Regions D and I are associated with the part of Broca's area, corresponding to BA 44; and Regions E and J are situated in the premotor and supplementary motor cortex, corresponding to BA 6.

### 2.4 Data Analysis

Optical data underwent a standardized preprocessing procedure using Homer2, a MATLAB‐based software (Huppert et al. 2009). Initially, the raw optical intensity data series were converted into changes in OD. We then applied a discrete wavelet transform to the data from each channel to remove motion artifacts using a tuning parameter (*α*) of 0.1. Next, a bandpass filter with cutoff frequencies between 0.01 and 0.2 Hz was applied to minimize slow drifts and high‐frequency noise.

Subsequently, the OD data were converted into concentration changes for HbO and HbR. To enhance the detection of neural activation, we utilized the correlation‐based signal improvement (CBSI) method on both HbO and HbR. The CBSI method is particularly effective at removing nonneuronal signal artifacts since it assumes an anticorrelation between neural activation signals of HbO and HbR. In contrast, motion artifacts tend to increase their positive correlation (Cui et al. 2010).

The HbO and HbR concentration changes from channels within the same region were averaged to obtain regional values. HbO was prioritized for further analysis due to its sensitivity to changes in fNIRS measurements (Moriguchi and Hiraki 2009). We converted the HbO concentrations into *z*‐scores using the mean and standard deviation of the HbO changes during the resting phase. We calculated the mean *z*‐scores for HbO separately across the three task blocks for each participant. A temporal window of interest was defined as 5–10 s after stimulus onset, based on the typical peak of the hemodynamic response occurring around 6 s afterward. We then averaged the HbO (*z*‐scores) within this time interval.

Finally, we conducted statistical analyses to identify regions with significant variations in cortical hemodynamics across the MR tasks. We utilized the Statistical Package for Social Science (SPSS v22; Chicago, IL, USA) for this analysis. We corrected the *p* values of the repeated ANOVAs using the false discovery rate (FDR) method, and a *p* value (FDR‐corrected) of less than 0.05 was considered statistically significant.

## 3 Results

### 3.1 Behavioral Results

Altogether, 62 participants (*M*_age_ *= *10.60, SD* = *0.35) completed the MR task in this study. Forty‐five boys (*M*_age_ = 10.60, SD = 0.38) and 17 girls (*M*_age_ = 10.60, SD = 0.255) were classified into boys' and girls’ groups. As shown in Table 2, the independent sample *t*‐test found no significant difference in the score of the MR1 task between boys (*M*_score_ = 23.40, SD* = *4.28) and girls (*M*_score_ = 24.06, SD = 3.071), *t =* −0.67, *p* = 0.505. Similarly, no significant difference was found in the score of the MR2 task between boys (*M*_score_ = 22.51, SD = 4.526) and girls (*M*_score_ *= *21.71, SD = 3.98), *t = *0.65, *p* = 0.522. To control the age effect, we conducted MANOVAs with MR1 and MR2 scores as the dependent variables, gender as the independent variable, and age as the covariate; the results were not significant, *F*s *<* 1.07, *p*s > 0.426, indicating no age effect.

**TABLE 2:** Behavioral results.

	Group	Mean	SD	*t* value	*p* value
MR1	Boys	23.40	4.277	−0.672	0.505
	Girls	24.06	3.071		
MR2	Boys	22.51	4.526	0.645	0.522
	Girls	21.71	3.981		

### 3.2 fNIRS Results

**FIGURE 5:**
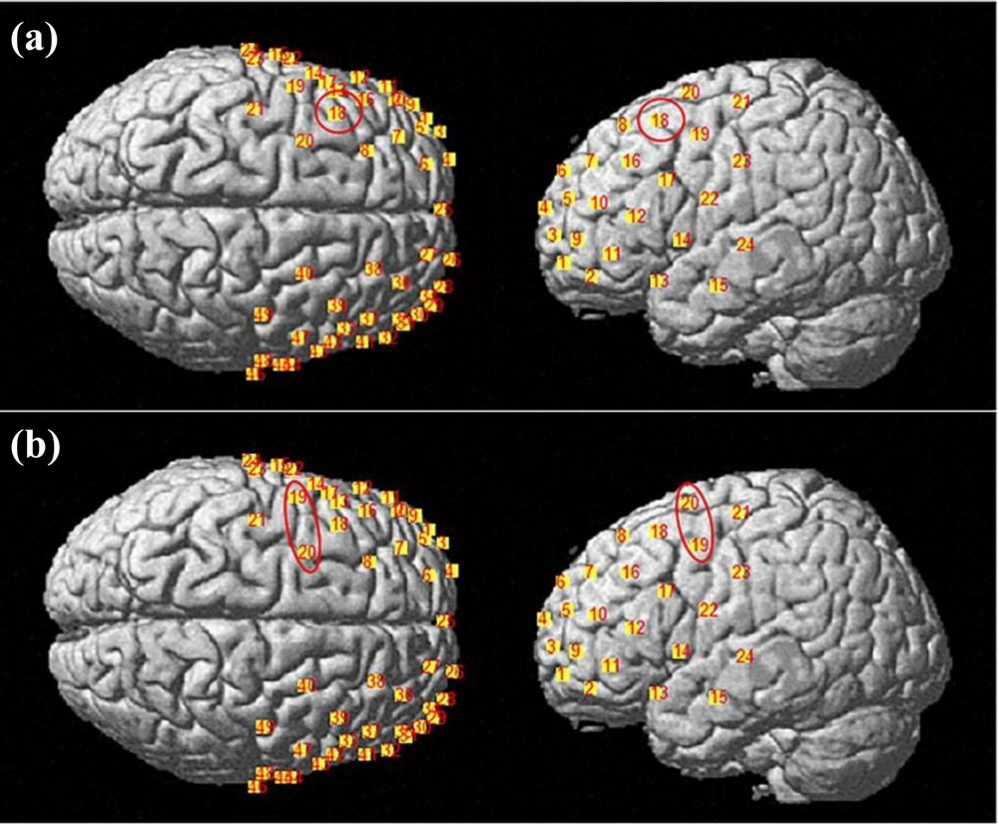
Regions that show the group's main effect in the mental rotation task marked on MNI space: (a) Region C (BA 9) contains Channel 18; (b) Region E (BA 6) contains Channels 19 and 20. The difference found in the group's main effect is circled in red.

**FIGURE 6:**
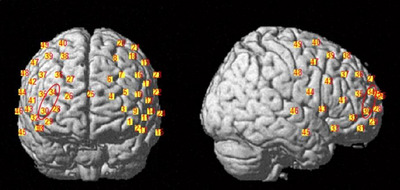
Regions that show Task and Group × Task interaction's primary effect in mental rotation tasks are marked on MNI space: Region G (BA 46), which contains Channels 30 and 34. The difference in Task and Group × Task's primary effect is circled in red.

**FIGURE 7:**
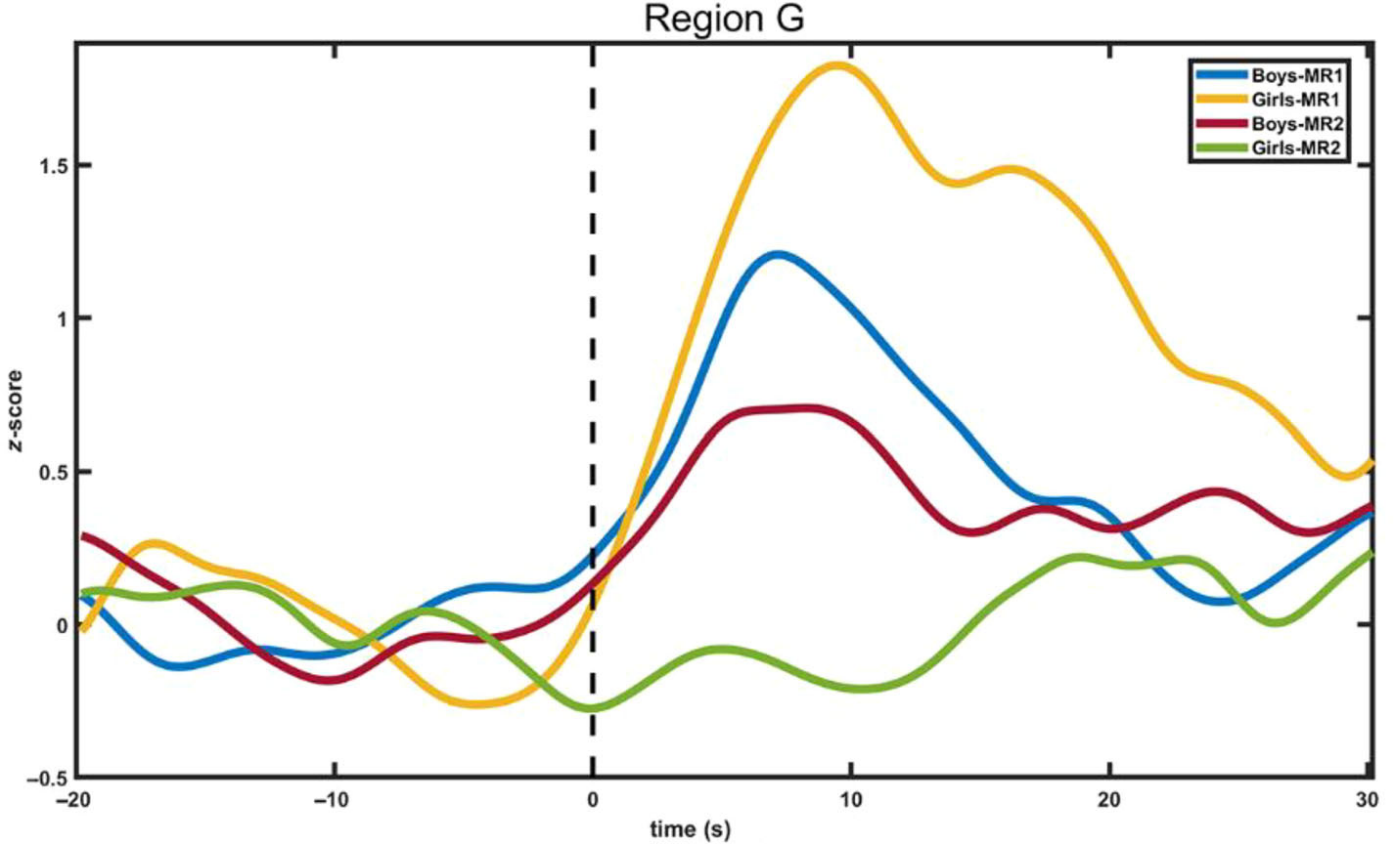
Temporal changes in the HbO concentration in Region G (BA 46) during the MR1 and MR2 tasks (boys and girls).

Brain activation of the boys and girls groups in the MR1 task and MR2 task was examined. First, a series of two sexes (boys, girls) X 2 tasks (MR1, MR2) repeated measures ANOVA models were performed in each region (Tables 3 and 4). A main effect of the group was evident in Region C (BA 9) (*F* = 8.413, *p* < 0.05) and Region E (BA 6) (*F* = 8.069, *p* < 0.05); channels in Region C and Region E were shown in Figure 5. A main effect of the task was evident in Region G (BA 46) (*F* = 23.601, *p* < 0.001). Channels in Region G are shown in Figure 6. A main effect of the Group X Task interaction was evident in Region G (BA 46) (*F* = 8.738, *p* < 0.05). Temporal changes in the HbO concentration in Region G are shown in Figure 7.

**TABLE 3:** The mean and SD of HbO changes (*z*‐score) in Boys (*N*1 = 45) and Girls (*N*2 = 17) during the task are shown.

	Group	Task	Mean	SD
Region A	Boys	MR1	0.706	1.383
		MR2	0.587	1.233
	Girls	MR1	0.654	1.174
		MR2	0.546	1.180
Region B	Boys	MR1	0.886	1.026
		MR2	0.795	1.047
	Girls	MR1	0.785	1.190
		MR2	0.508	1.204
Region C	Boys	MR1	1.338	1.388
		MR2	0.812	1.218
	Girls	MR1	0.515	1.120
		MR2	−0.027	1.313
Region D	Boys	MR1	0.799	1.753
		MR2	0.393	1.491
	Girls	MR1	0.636	1.291
		MR2	0.152	1.329
Region E	Boys	MR1	0.716	1.430
		MR2	0.564	1.080
	Girls	MR1	0.177	0.923
		MR2	−0.488	1.307
Region F	Boys	MR1	0.876	1.403
		MR2	0.654	1.224
	Girls	MR1	0.498	1.166
		MR2	0.099	1.211
Region G	Boys	MR1	1.128	1.521
		MR2	0.691	1.144
	Girls	MR1	1.649	1.480
		MR2	−0.146	1.564
Region H	Boys	MR1	1.120	2.181
		MR2	0.522	1.312
	Girls	MR1	0.230	1.330
		MR2	−0.047	1.435
Region I	Boys	MR1	0.577	1.538
		MR2	0.513	1.425
	Girls	MR1	0.295	1.482
		MR2	−0.145	1.765
Region J	Boys	MR1	0.552	1.493
		MR2	0.785	1.296
	Girls	MR1	0.511	1.020
		MR2	0.627	1.359

**TABLE 4:** Tests of within‐subjects effects and between‐subjects effects in HbO.

	Group		Task		Group × Task	
	F	Sig.	F	Sig.	F	Sig.
Region A	0.033	0.856	0.197	0.659	0.001	0.983
Region B	0.627	0.720	0.972	0.468	0.249	0.983
Region C	8.413	0.030*	5.540	0.110	0.001	0.983
Region D	0.401	0.755	2.134	0.248	0.016	0.983
Region E	8.069	0.030*	3.744	0.193	1.480	0.983
Region F	2.425	0.266	2.199	0.248	0.179	0.983
Region G	0.239	0.782	23.601	0***	8.738	0.040*
Region H	3.635	0.203	2.193	0.248	0.292	0.983
Region I	2,320	0.266	0.690	0.511	0.385	0.983
Region J	0.143	0.785	0.388	0.595	0.044	0.983

*** *p* < 0.05.

Next, a set of independent‐sample *t*‐tests was conducted to analyze the difference in brain activation in Region C (BA 9), Region E (BA 6), and Region G (BA 46) between the boys and girls. As shown in Tables 5 and 7, the results indicated a significant sex difference in Region C (BA 9) (*t* = 2.409, *p* < 0.05) in the MR1 task, with the boys (*M* = 1.338, SD = 1.388) having significantly more HbO increase than the girls (*M* = 0.515, SD = 1.120). A significant sex difference in Region C (BA 9) (*t* = 2.369, *p* < 0.05), Region E (BA 6) (*t* = 3.230, *p* < 0.05), and Region G (BA 46) (*t* = 2.317, *p* < 0.05) in the MR2 task, with boys (*M*_Region C_ = 0.812, SD_Region C_ = 1.218, *M*_Region E_ = 0.564, SD_Region E_ = 1.080, *M*_Region G_ = 0.691, SD_Region G_ = 1.144) having significantly more HbO increase than the girls (*M*_Region C_ = −0.027, SD_Region C_ = 1.313, *M*_Region E_ = −0.488, SD_Region E_ = 1.307, *M*_Region G_ = −0.146, SD_Region G_ = 1.564).

**TABLE 5:** Independent samples in Region C (BA 9), Region E (BA 6), and Region G (BA 46).

Task	Region	*t* value	*p* value
MR1	C	2.409	0.020*
	E	1.741	0.088
	G	−1.221	0.230
MR2	C	2.369	0.021*
	E	3.230	0.002**
	G	2.317	0.024*

* *p* < 0.05.

** *p* < 0.01.

Third, paired *t*‐tests were performed on the mean HbO to examine the between‐task differences between boys and girls at Region G (BA 46). As shown in Tables 6 and 7, the results revealed that girls exhibited significant differences between the MR1 task and MR2 task at Region G (BA 46) (*t* = 3.920, *p* = 0.001), with significantly more HbO increase in the MR1 task (*M* = 1.649, *SD* = 1.480) than in the MR2 task (*M* = −0.146, *SD* = 1.564). No significant differences were found between MR1 and MR2 tasks in Region G in the boys’ group.

**TABLE 6:** Paired samples *t*‐test in Region G (BA 46).

Group	Region	*t* value	*p* value
Boys	G	1.952	0.057
Girls	G	3.920	0.001**

** *p* < 0.01.

**TABLE 7:** Mean and SD of HbO changes (*z*‐score) in Region C (BA 9), Region E (BA 6), and Region G (BA 46).

		Boys		Girls		*t* value
		Mean	SD	Mean	SD	
Region C (BA 9)	MR1	1.338	1.388	0.515	1,120	2.409*
	MR2	0.812	1.218	−0.027	1.313	2.369*
Region E (BA 6)	MR1	0.716	1.430	0.177	0.923	1.741
	MR2	0.564	1.080	−0.488	1.307	3.230**
Region G (BA 46)	MR1	1.128	1.521	1.649	1.480	−1.221
	MR2	0.691	1.144	−0.146	1.156	2.317*
	*t* value	1.952		3.920**		

* *p* < 0.05.

** *p* < 0.01.

### 3.3 Brain Behavior Correlation Results

The Pearson correlation coefficient was employed to examine the relationship between the mean HbO levels of each region and task scores. In boys, the mean HbO of Region E exhibited a significant positive correlation with task scores in MR2 (*r* = 0.351, *p *= 0.018). In contrast, the other regions for boys and all areas for girls did not demonstrate significant correlations between behavioral and HbO scores (*r*s < 0.351, *p*s > 0.05).

## 4 Discussion

This research was conducted to explore the performance differences between boys and girls (sex effect) and between MR1 and MR2 (task effect). The behavioral findings, however, demonstrated null results for the sex and task effects; in contrast, the fNIRS evidence indicated both significant sex and task effects, with girls performing better in MR1 and boys performing better in MR2. This section will discuss these conflicting and interesting findings, their limitations, and implications for future human behavioral studies.

### 4.1 Behavioral Evidence Demonstrated Null Results

The question of sex differences in MR proficiency has long captivated academic inquiry. Numerous behavioral studies and meta‐analyses have suggested that men generally outperform women in this domain (Fargier et al. 2022; Hyde 2005; Rahe and Jansen 2022; Voyer et al. 1995; Voyer et al. 2020). However, the findings of this study challenge this prevailing notion, as both boys and girls who were left behind children living in rural China performed the MR1 and MR2 tasks at notably similar levels. As a result, the behavioral evidence presented here suggests an absence of sex differences in MR proficiency. Additionally, the lack of significant performance differences between the MR1 and MR2 tasks supports the conclusion that no task effect exists.

Nevertheless, it is crucial to acknowledge that behavioral studies are not immune to the risk of Type II errors, where actual effects may go undetected. Therefore, neuroimaging evidence is essential for a more comprehensive understanding of potential sex and task differences in brain activation. Comparing this behavioral evidence with neuroimaging ones will provide valuable insights and help clarify whether cognitive processes differ between sexes, even when behavioral outcomes appear similar.

### 4.2 fNIRS Evidence Demonstrated Significant Sex Differences

This study revealed significant sex differences in brain activation during MR2 (letter rotation), with boys exhibiting greater activation than girls in BA 6, 9, and 46. These prefrontal regions are critical for higher‐order cognitive functions. BA6, encompassing the premotor cortex and supplementary motor area, is involved in planning and executing movements (Wu et al. 2020; Yang et al. 2020). BA9, located in the dorsolateral and medial prefrontal cortex, contributes to working memory, attentional control, and cognitive flexibility (Petrides 2005). BA46, situated in the DLPFC anterior to BA6, plays a key role in working memory maintenance, sustained attention, and central executive functions (Petrides 2005).

Previous fNIRS research has linked BA6 and BA46 to cognitive shifting and MR (Li et al. 2021; Wu et al. 2020; Yang et al. 2020). While BA9 has been identified as a neural correlate of MR in preschoolers, studies also suggest its involvement in inhibiting automatic responses and enhancing cognitive flexibility during MR tasks (Yang et al. 2020). The increased activation observed in boys during the letter rotation task (MR2) could indicate a greater reliance on these cognitive resources. This might reflect task‐specific demands related to familiarity with the English alphabet.

However, interpreting these sex differences solely through the lens of cognitive demand overlooks the potential influence of stereotype threat. Stereotype threat theory posits that awareness of negative stereotypes about one's group can impair performance (Steele and Aronson 1995). While the stereotype of males possessing superior spatial skills might suggest boys would be less susceptible, the added layer of letter recognition, stereotypically associated with a female advantage in language skills, introduces a potential confound. Boys might experience stereotype threat related to this aspect of the task, leading to increased cognitive effort and correspondingly higher activation in BA6, 9, and 46. This is further complicated by the finding that boys also demonstrated higher activation in BA9 during MR1, a task not involving letter recognition and arguably favoring boys according to spatial stereotypes. This inconsistency highlights the complexity of interpreting these results and underscores the need for further investigation.

Future research must incorporate methodologies that mitigate stereotype threat and directly examine its neural correlates to disentangle the effects of stereotype threat from genuine sex‐based differences in brain function. For example, comparing performance and brain activation under different stereotype‐relevant instructions or manipulating the salience of gender stereotypes could provide valuable insights. While this study raises important questions about the relationship between brain regions, cognitive tasks, and sex, a more nuanced approach that accounts for stereotype threat is crucial to elucidating the underlying mechanisms driving these observed differences.

### 4.3 fNIRS Evidence Demonstrated Significant Task Differences

This study identified significant task effects within the female group, revealing greater brain activation in BA46 during the MR1 task compared to MR2. In contrast, no such task effects were observed in the male group. BA46, located between BA10 and BA45, is part of the DLPFC, which includes both BA46 and BA9. The DLPFC is known to be involved in high‐level cognitive tasks and decision‐making processes. While the specific functions and boundaries of BA9 and BA46 can vary among individuals, both are generally recognized as essential components of the neural circuitry that support executive functions, attention regulation, and working memory management. The observed difference in this study suggests that the cognitive demands of the rotating figures task require more significant hemodynamic increases in brain activation for girls, indicating that task nature may significantly influence brain engagement levels. This finding highlights a complex interaction between task demands and sex‐specific physiological responses in the brain.

In addition, the fNIRS evidence in this study demonstrated an interaction between sex and task during MR tasks. Notably, although girls showed superior ability in rotating letters compared to boys, they required more brain activation to complete figure rotations than their male counterparts. This suggests inherent sex‐specific physiological differences in cognitive processing, with task‐related implications for approaching MR tasks.

Lastly, the conflicting results between behavioral outcomes and fNIRS evidence imply that traditional behavioral studies may accept a false and nonsignificant conclusion, leading to Type II errors. Therefore, future investigations into human behavior should consider integrating neuroimaging methodologies to mitigate the risk of producing misleading results and to gain a deeper understanding of cognitive processing differences.

## 5 Conclusions, Limitations, and Implications

In conclusion, this exploration of cognitive skill MR among Chinese primary students yielded intriguing findings, particularly emphasizing the effects of sex‐task interaction. While behavioral evidence reflected no sex or task effects in MR performance, the fNIRS evidence revealed significant sex and task effects. Boys showcased heightened brain activation in BA 6, 8, and 9 than girls. Girls, however, demonstrated increased activation during the figure rotation tasks. This sex‐task interaction underscores the complex relationship between sex, task, and cognitive processing in children.

The present study, while valuable, has several limitations that warrant attention. First, this study sampled the left‐behind children (low SES) in a rural Chinese primary school. In the future, recruiting a more diverse sample across different demographic backgrounds would strengthen the generality of findings. Second, this study employed 2D stimuli, which limited our assessment of MR in left‐behind children who might not be familiar with 3D stimuli. Future research on ordinary students should incorporate 3D tasks for a more comprehensive analysis. Additionally, the whole grade sampling and random assignment of groups resulted in an imbalanced gender representation, which could be addressed in future studies for more robust conclusions. Finally, as this study did not explore cross‐cultural perspectives, it cannot assess the impact of gender stereotypes on MR performance, a crucial area for future research, given evidence that preschool‐aged children have explicit stereotypes linking spatial abilities to boys.

Nevertheless, this study enhances our understanding of how sex and task variables influence the brain processes underlying MR tasks. First, it reveals a complex interplay between sex and task performance that calls for a dedicated, context‐driven, and culturally informed approach to neuropsychological research. The findings highlight that sex differences manifest distinctly across various MR tasks, suggesting a need for more targeted, nuanced research agendas. Second, this study deepens our understanding of the effects of sex‐task interaction and reinforces the argument for integrating neuroimaging techniques in cognitive research. Our findings signal the importance of adopting reliable neuroimaging methodologies to account for the underlying brain differences contributing to mental processing. This study lays the groundwork for exploring new avenues in human behavioral studies by illuminating these nuances. When traditional behavioral evidence suggests a nonsignificant result, neuroimaging can reveal the subtle differences in brain activation associated with behavioral performance. This underscores the critical role of neuroimaging in providing a more comprehensive understanding of cognitive processes and their variations across different populations.

## Author Contributions

**Dandan Wu**: conceptualization, investigation, methodology, visualization, project administration, supervision, data curation. **Jinfeng Yang**: investigation, writing–original draft, methodology, writing–review and editing, formal analysis, data curation. **Zhi Hong Wan**: funding acquisition, conceptualization, supervision, resources. **Yining Shen**: investigation, data curation. **Qianming Liu**: investigation, data curation. **Jinghui Zhang**: investigation, data curation. **Simin Cao**: investigation, data curation. **Hui Li**: conceptualization, funding acquisition, methodology, writing–review and editing, project administration, resources, supervision.

## Ethics Statement

This study was approved by The Education University of Hong Kong (ethical clearance reference no.: EDUHK2021‐2022‐0129).

## Conflicts of Interest

The authors declare no conflicts of interest.

### Peer Review

The peer review history for this article is available at https://publons.com/publon/10.1002/brb3.70358

## Data Availability

The authors will provide data upon a written request.
